# CDA-2, a Urinary Preparation, Inhibits Lung Cancer Development through the Suppression of NF-kappaB Activation in Myeloid Cell

**DOI:** 10.1371/journal.pone.0052117

**Published:** 2012-12-17

**Authors:** Xuan Wang, Cui-Min Jiang, Hai-Ying Wan, Jun-Lu Wu, Wen-Qiang Quan, Robert Bals, Kai-Yin Wu, Dong Li

**Affiliations:** 1 Department of Pharmacy, Putuo People’s Hospital, Shanghai, China; 2 Department of Clinical Laboratory, Tongji Hospital of Tongji University, Shanghai, China; 3 Department of Internal Medicine V – Pulmonology, Allergology, Respiratory Intensive Care Medicine, Saarland University Hospital, Homburg, Germany; 4 Institute of Pathology, Charité University Hospital, Berlin, Germany; Mie University Graduate School of Medicine, Japan

## Abstract

CDA-2 (cell differentiation agent 2), a urinary preparation, has potent anti- proliferative and pro-apoptotic properties in cancer cells. However, the mechanisms of tumor inhibitory action of CDA-2 are far from clear, and especially there was no report on lung cancer. Here we demonstrate that CDA-2 and its main component phenylacetylglutamine (PG) reduce the metastatic lung tumor growth, and increases survival time after inoculation with Lewis lung carcinoma (LLC) cells in a dose-dependent manner in C57BL6 mice. Proliferative program analysis in cancer cells revealed a fundamental impact of CDA-2 and PG on proliferation and apoptosis, including Bcl-2, Bcl-XL, cIAP1, Survivin, PCNA, Ki-67 proteins and TUNEL assays. CDA-2 and PG significantly reduced NF-κB DNA-binding activity in lung cancer cells and in alveolar macrophages of tumor bearing mice and especially decreased the release of inflammatory factors including TNFα, IL-6, and KC. Furthermore, CDA-2 and PG decrease the expressions of TLR2, TLR6, and CD14, but not TLR1, TLR3, TLR4, and TLR9 in bone-marrow-derived macrophages (BMDM) of mice stimulated by LLC-conditioned medium (LLC-CM). Over-expressing TLR2 in BMDM prevented CDA-2 and PG from inhibiting NF-κB activation, as well as induction of TNFα and IL-6. TLR2:TLR6 complexes mediate the effect of NF-κB inactivation by CDA-2. In conclusion, CDA-2 potently inhibits lung tumor development by reduction of the inflammation in lung through suppression of NF-κB activation in myeloid cells, associating with modulation of TLR2 signaling.

## Introduction

Lung cancer is the leading cause of cancer deaths in the world, causing more than one million deaths worldwide [1]. Despite advances in early detection and standard treatment, lung cancer is often diagnosed at an advanced stage and has a poor prognosis. Therefore, prevention and treatment of lung cancer are the focus of intensive current research [2].

CDA-2 (cell differentiation agent 2) is a urinary preparation that isolated from healthy human urine in China. It is a novel multifunctional drug that is useful for both the prevention and treatment of several tumors, including leukemia, breast cancer, liver cancer, and pheochromocytoma, in preclinical investigations [3]–[5]. However, the mechanisms of tumor inhibitory action of CDA-2 are far from clear, and especially there was no report on lung cancer. CDA-2 contains multiple active components, including phenylacetylglutamine (PG) (41%), benzoyl glycocoll (35%), peptides (MW 400–2800) (17%), 4-OH-phenylacetic acid (6%), and 5-OH-indoleacetic acid (1%), which with different mechanisms of anticancer [5]. Although tumor inhibition may be attributed to these components, PG is likely to be a major tumor inhibitory component [3]. Phase I/II/III clinical trials of CDA-2 have been completed in China in 2003. In August 2004, the State Drug Administration (SDA) of China approved the use of CDA-2 as an anticancer drug in solid tumors. Although CDA-2 was suggested to contribute to tumor inhibition through the up-regulation of peroxisome proliferator-activated receptor-γ (PPAR-γ) and repression of PI3/Akt signaling pathway in tumor cells, the tumor-inhibiting effect of CDA-2 was so far mainly demonstrated in cancer cells and its action in tumor microenvironments, especially to immune/inflammatory cells in tumor stroma, has not been critically evaluated [6], [7].

NF-κB is a key coordinator of inflammatory and immune response and has recently been found to play a pivotal role in carcinogenesis of a number of cancers including lung or colon carcinoma [8], [9]. It is noteworthy that the pro-inflammatory cytokines and chemokines have been linked to carcinogenic processes in humans and mice, and are regulated by the NF-κB pathway. For example, NF-κB-driven cytokine production by myeloid cells (e.g., mature macrophages, dendritic cells, and neutrophils) such as TNF-α and IL-6 are required for lung tumor growth [9]. In a mouse model of colitis-associated cancer (CAC), IKKβ was deleted in myeloid cells (leading to decreased NF-κB activity), tumor size was considerably smaller compared to controls and expression of pro-inflammatory cytokines, such as TNFα, IL-6, and IL-1, was also markedly reduced [10]. Thus in myeloid cells, NF-κB activation promotes tumor growth. This effect is mainly due to enhanced tumor cell proliferation via the production of TNFα, IL-6, and other cytokines that are regulated by the NF-κB pathway in myeloid cells [10], [11].

Here, we report our recent work concerning the tumor suppression and the molecular mechanisms of CDA-2 and its main constituent, PG, to lung cancer. We used experimental murine lung cancer models in which CDA-2 and PG reduces lung tumor growth, and demonstrated that NF-κB inactivation in myeloid cells is responsible for CDA-2-induced tumor regression. We found that the inhibition of TLR-2 signaling is a key mechanism of CDA-2-induced NF-κB inactivation. Our results suggest a novel theory for cancer therapy by CDA-2, based on the inhibition of NF-κB in myeloid cells of tumor microenvironments.

## Materials and Methods

### Cell Culture

The mouse Lewis lung carcinoma (LLC) cells were obtained from the American Type Culture Collection and cultured in Dulbeccos’s modified Eagles medium (DMEM, Hyclone laboratories. Inc, South, Utah, USA) supplemented with 10% fetal calf serum (FCS) (Invitrogen, Grand Island, NY, USA), 100 U/mL penicillin, and 100 U/mL streptomycin (Hyclone laboratories. Inc, South, Utah, USA). Cell cultures were performed at 37°C in humidified air with 5% CO_2_.

### Animals

Female C57BL/6 mice were obtained from the National Rodent Laboratory Animal Resource (Shanghai Branch, PRC) and maintained under a pathogen-free Central Animal Facility of the Tongji University. This study was carried out in strict accordance with the recommendations in the Guidelines for the Care and Use of Laboratory Animals of the National institutes of Health. All animal experiments were approved by the Tongji University Ethics Committee on the Use and Care of Animals.

All surgery was performed under sodium pentobarbital anesthesia, and all efforts were made to minimize suffering.

### Generation of Lung Cancer Model in Mice and Treatment of CDA-2 and PG

A lung cancer metastasis model in C57BL/6 mice was generated by intravenous injection of LLC cells. Briefly, subconfluent LLC cells or A549 cells were harvested and passed through a 40 µm cell strainer (BD Biosciences, Bedford, MA, USA), washed three times with PBS, resuspended in serum free DMEM and injected at a concentration of 2×10^5^ LLC cells per mouse into the tail vein. After14 days, mice were injected intraperitoneally (i.p.) with 500 mg/kg, 1000 mg/kg, and 2000 mg/kg CDA-2 (kindly supplied by Ever Life Pharmaceutical Co. Ltd. Hefei, Anhui, China) or 200 mg/kg, 400 mg/kg, and 800 mg/kg PG (Sigma Aldrich, Steinheim, Germany) in PBS or PBS alone once everyday for 10 days.

### Evaluation of Lung Tumors

At designated time points, mice were killed, and their lungs were removed, weighed, and histologically examined. Some mice were kept until death and survival data were obtained. Lung tumour nodules were microdissected using an 18 G needle under a microscope for protein analysis. Tumor multiplicity and maximal sizes were determining as described [9]. In brief, whole tumor-bearing lungs were manually inflated with and fixed in 4% paraformaldehyde and embedded. Paraffin-embedded lungs were serially sectioned at 350 µm and histologically examined with hematoxylin and eosin (H&E).

### Immunohistochemical Analyses

Immunohistochemistry was performed as described before [12]. Mouse anti-Ki-67 (Abcam, Cambridge, UK) was used as primary antibody. Secondary antibody incubation and staining were performed using the EnVision®+ System–HRP (AEC) kit (Dako, Carpinteria, CA, USA) according to manufacturer’s recommendations. For TUNEL assay, a DeadEnd™ Colorimetric TUNEL System (Promega) was used according to the manufacturer’s recommendations. The number of Ki-67 or TUNEL-positive tumor cells and the total number tumor cells was measured in six microscopic fields of randomly selected tumors and then the mean value was calculated as the percentage of Ki-67 or TUNEL-positive tumor cells.

### Western Blotting

Lung tumor nodules were carefully microdissected using an 18 G needle from lungs under a microscope. For total protein isolation, 10 mg tumor nodule were homogenized in the 500 µl cell lysis buffer (Cell Signalling Technology, Danvers, MA, USA) containing 5 mM PMSF and protease inhibitors using rotor-stator homogenizer. Western blot analysis was performed as described earlier [13]. Briefly, total protein extracts were loaded on 10% SDS-polyacrylamide gels, subjected to electrophoresis, and blotted onto Hybond-C Extra membranes (Amersham Bioscience, Buckinghamshire, United Kingdom). The primary antibodies included: mouse anti-Bcl-XL, mouse anti-Bcl-2 (both from Santa Cruz Biotechnology, Santa Cruz, CA, USA); mouse anti-PCNA, rabbit anti-cIAP1, rabbit anti-Survivin (all three from Abcam, Cambridge, UK); mouse anti-β-actin (Sigma Aldrich, Steinheim, Germany). HRP-conjugated goat anti-rabbit (Santa Cruz Biotechnology) or rabbit anti-mouse were used as secondary antibody (Dako, Glostrup, Denmark).

### Bronchoalveolar Lavage (BALF) Leukocyte Counts and Alveolar Macrophages Isolation

BALF was determined as described previously [14]. Percentages of leukocyte subpopulations were determined by counting 100 leukocytes in a randomly selected portion of the cytospin slide. The total number of leukocytes in the BALF was determined by using a hemocytometer (Beckman Coulter, Miami, FL, USA). Alveolar macrophages were isolated for EMSA as previously described [15]. Briefly, the BALF was seed in DMEM and maintained in cell culture dish. Cells were incubated at 37°C in humidified air with 5% CO2 for 1 hour. The dish then was washed 3 times with PBS, and the adherent cells, predominantly macrophages, were collected for nuclear protein extract.

### Electrophoretic Mobility Shift Assay (EMSA)

Nuclear proteins were isolated from lung tumors and alveolar macrophages using Nuclear Extract Kit (Active Motif, Carlsbad, CA, USA) and NF-κB DNA binding activity was measured by EMSA as described [13]. Briefly, nuclear proteins were incubated with [^32^p]–labeled double-stranded NF-κB consensus probe (Promega) at room temperature for 30 min. DNA–protein complexes were resolved on 4% polyacrylamide gels equilibrated in 0.5× TBE under 300V. Gels were dried and exposed to Hyperfilm ECL (Amersham Bioscience) at −80°C and developed using Kodak film (Eastman Kodak, Rochester, N.Y., USA).

### Cytokines ELISA Assay

BALF samples were prepared as described above. TNFα, IL-6, and KC were measured by commercially available sandwich-type ELISA (R&D Systems, Minneapolis, MN, USA), following the manufactures’ instructions.

### Mouse Bone Marrow-derived Macrophages (BMDMs) Isolation and Luciferase Reporter Assay

Cells from the bone marrow of C57BL6 mice were cultured in DMEMs medium (10% FCS) supplemented with 10 ng/ml recombinant mouse M-CSF (eBioscience, San Diego, CA, USA) for 7 days to allow differentiation to macrophages. Adenoviral constructs encoding the full-length of cDNA TLR2 were created using the AdEasy system as previously described [16], [17]. The NF-κB luciferase adenovirus plasmid pNF-κB-Leu (BD Clontech) containing multiple copies of NF-κB consensus sequence to monitor NF-κB activation. BMDMs (1×10^5^ per well of 12-well plates) were infected with the TLR2 adenoviral plasmids and control plasmids, after 5 hours, cells were infected again with luciferase adenovirus plasmid pNF-κB-Leu. Followed by 24 hours incubation, the infected cells were stimulated for 24 hours with LLC-CM or/and CDA-2, and then were lysed and luciferase reporter gene activity was determined by the Luciferase Reporter assay (Promega, Madison, WI, USA).

### LLC Conditioned Medium

Conditioned medium was collected from LLC cells incubated in serum-free DMEM (SFM) for 24 h, and filtered through a 0.2 µm filter. Conditioned medium samples were added to BMDMs for 24 h, after which TLRs genes expression were assayed.

### RNA Isolation and Real-time PCR

Total lung tissue and BMDMs RNA were prepared with RNeasy plus mini kit (Qiagen, Santa Clarita, CA, USA) according to manufacturer’s recommendation. Real time PCR reaction mixtures have been described previously [18]. Briefly, cDNA was synthesized by reverse transcription reaction using the First Strand cDNA synthesis kit (Invitrogen). Real-time PCR was performed using the QPCR SYBR Green Mix (Bio-Rad, Hercules, CA, USA) on an AB 7300 Real time PCR system machine (AB Applied Biosystems, Singapore). The following PCR primers were used: mouse β-actin, 5′-AGCCTCGCCTTTGCCGA-3′ and 5′-CTGGTGCCTGGGGCG-3′; mouse Il1β, 5′-CAACCAACAAGTGATATTCTCCATG-3′ and 5′-GATCCACACTC TCCAGCTGCA-3′; mouse Il6, 5′-CCGGAGAGGAGACTTCACAG-3′ and 5′-TCC ACGATTTCCCAGAGAAC-3′;mouse Tnfα, 5′-AGCCCCCAGTCTGTATCCTT-3′ and 5′-CTCCCTTTGCAGAACTCAGG-3′; mouse Kc, 5′-CTTGGGGACACCTTT TAGCA-3′ and 5′-GCTGGGATTCACCTCAAGAA-3′; mouse Mip1, 5′-TGGAG CTGACACCCCGAC-3′ and 5′-ACGATGAATTGGCGTGGAA-3′; mouse Mcp1, 5′-GCAGGTCCCTGTCATGCTTC-3′ and 5′-TCCAGCCTACTCATTGGGATCA-3′; mouse Tlr2, 5′- TGGTGTCTGGAGTCTGCTGTG -3′ and 5′- CGCTCCGTACGAA GTTCTCAG -3′; Tlr6, 5′- CAACTTAACGATAACTGAGAG -3′ and 5′- CCAGAG AGGACATATTCTTAG -3′; CD14, 5′- ACA TCT TGAACC TCC GCA AC -3′ and 5′- AGGGTTCCTATCCAGCCTGT -3′. Specificity of RT-PCR was controlled by ‘‘no reverse transcription’’ controls and melting curve analysis. Quantitative PCR results were obtained using the ΔΔCT (cycle threshold) method. Data were normalized to β-actin levels in each sample.

### Statistical Analysis

Values are displayed as mean plus or minus SEM. Comparisons between groups were analyzed by the t test (two-sided) or ANOVA for experiments with more than two subgroups or Kaplan-Meier survival analysis. Results were considered statistically significant for P values less than 0.05.

## Results

### CDA-2 Decreases Lung Tumor Growth in Mice Tumor Models

**Figure 1:**
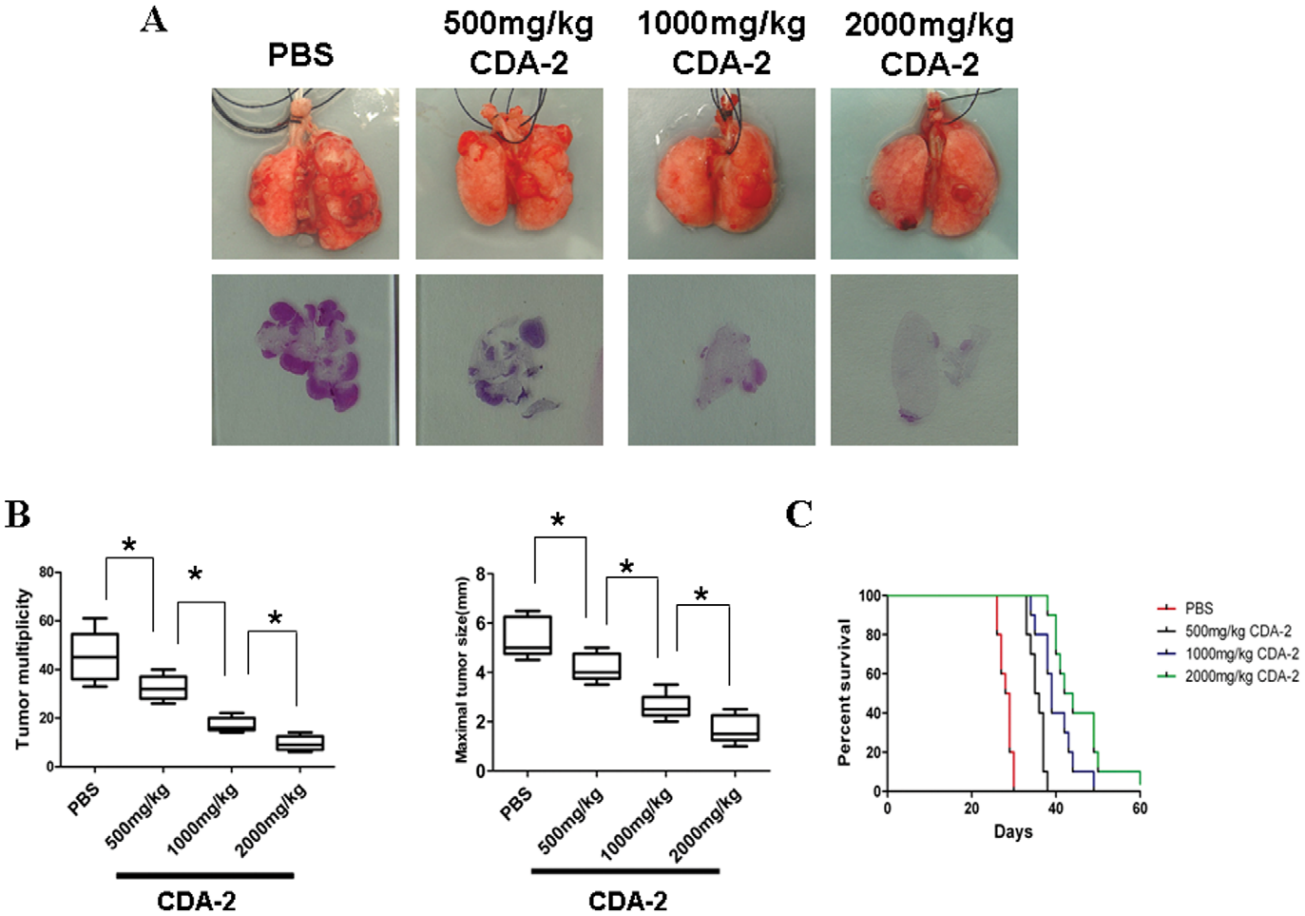
CDA-2 reduces development of lung tumor in mice. (A) Lung appearance (up) and histology (H&E stain; down) in LLC inoculated C57/BL6 mice 10 days after CDA-2 treatment with indicated doses. 2×10^5^ LLC cells were intravenously injected into sex-matched C57/BL6 mice by tail vein, 14 days later, mice were treated with PBS or CDA-2 for 10 days, at day 25, the lungs were removed. (B) Lung tumor multiplicity and maximal tumor sizes were determined by serial sectioning at 350 µm intervals. Results are mean ± SEM, n = 5, significant difference, * p<0.05. (C) Survival curves of mice (p<0,001; Log-rank test for statistic analysis; n = 10).

**Figure 2:**
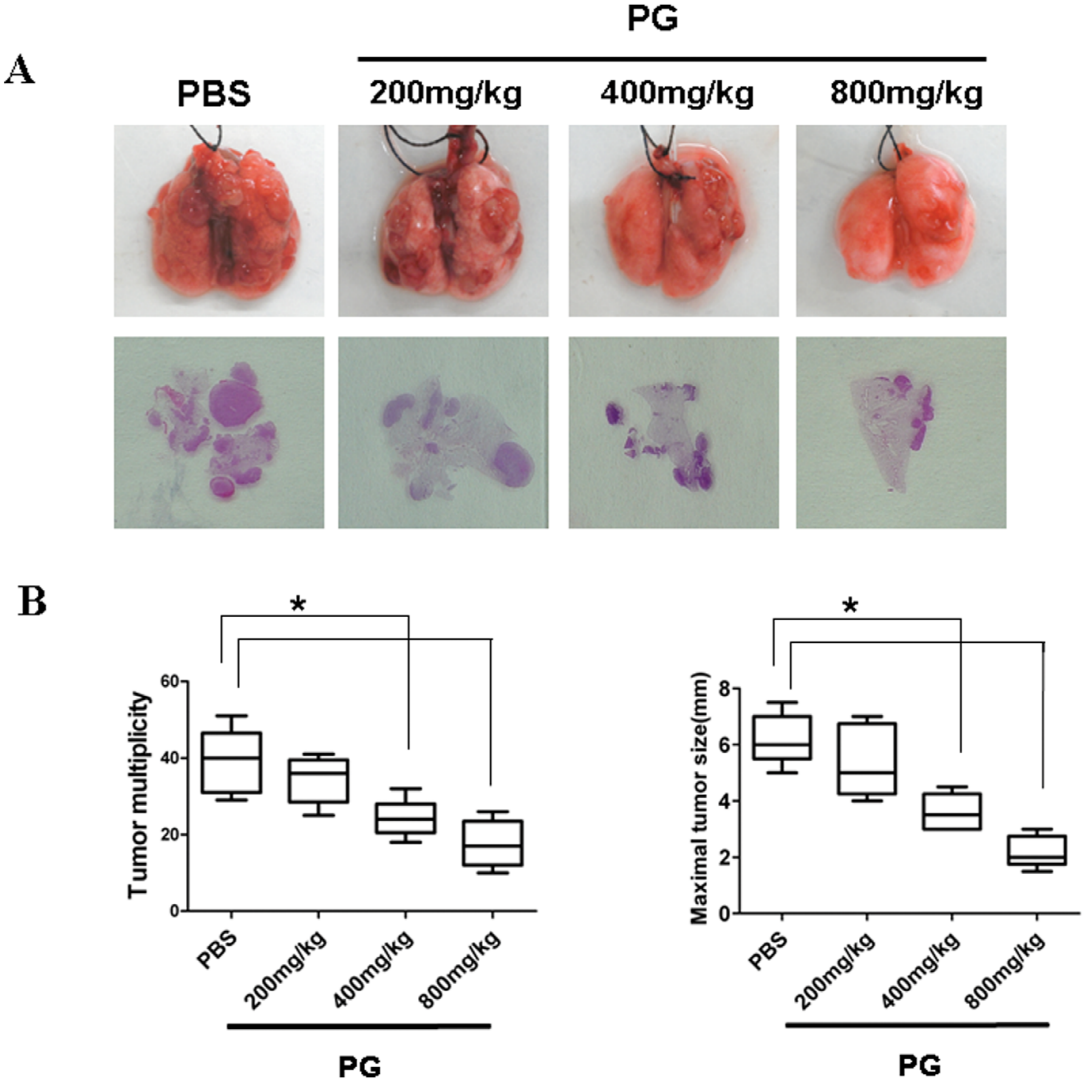
PG inhibits lung tumor promotion. (A) Lung appearance (up) and histology (H&E stain; down) in LLC inoculated C57/BL6 mice 10 days after PG treatment with indicated doses. 2×10^5^ LLC cells were intravenously injected into sex-matched C57/BL6 mice by tail vein, 14 days later, mice were treated with PBS or PG for 10 days, at day 25, the lungs were removed. (B) Lung tumor multiplicity and maximal tumor sizes were determined as in Figure 1B. Results are mean ± SEM, n = 5, significant difference, * p<0.05.

To investigate the effect of CDA-2 and its main component PG on growth of lung tumor, tumors were generated by intravenous injection of 2×10^5^ LLC cells in C57BL6 mice. After14 days, mice were injected intraperitoneally (i.p.) with 500 mg/kg, 1000 mg/kg, and 2000 mg/kg CDA-2 or 200 mg/kg, 400 mg/kg, and 800 mg/kg PG in PBS or PBS alone once everyday for 10 days. Mice were sacrificed, and their tumor multiplicity and maximal tumor sizes of lung tumors were evaluated. By contrast with control, administration of CDA-2 to the mice significantly reduced lung tumor multiplicity and maximal tumor sizes (Fig. 1A,B). H&E staining confirmed the massive reduction of tumor load in CDA-2-treated mice. (Fig. 1A). There are also significant differences in lung tumor burdens after different doses of CDA-2 administration indicating CDA-2 inhibited metastatic tumor growth in a dose-dependent manner (Fig. 1A,B). Similarly, PG also had a significant inhibitory effect on metastatic growth of lung tumor, as revealed by macroscopy and microscopy examination (Fig. 2A,B). We also assessed the survival times of tumor injected mice that were treated with CDA-2 by Kaplan-Meier survival analysis. Mice survival times were assessed and showed that the life spans of tumor-bearing mice were prolonged when given different concentration of CDA-2 treatment (Fig. 1C). There also had significant difference in the survival rate of different concentration CDA-2-treated tumor-bearing mice (Fig. 1C). These results confirm those obtained by examining tumor multiplicity, maximal tumor sizes, H&E staining from lung metastatic carcinomas models.

### CDA-2 Reduced Proliferation and Induced Apoptosis in Lung Cancer Cells

**Figure 3:**
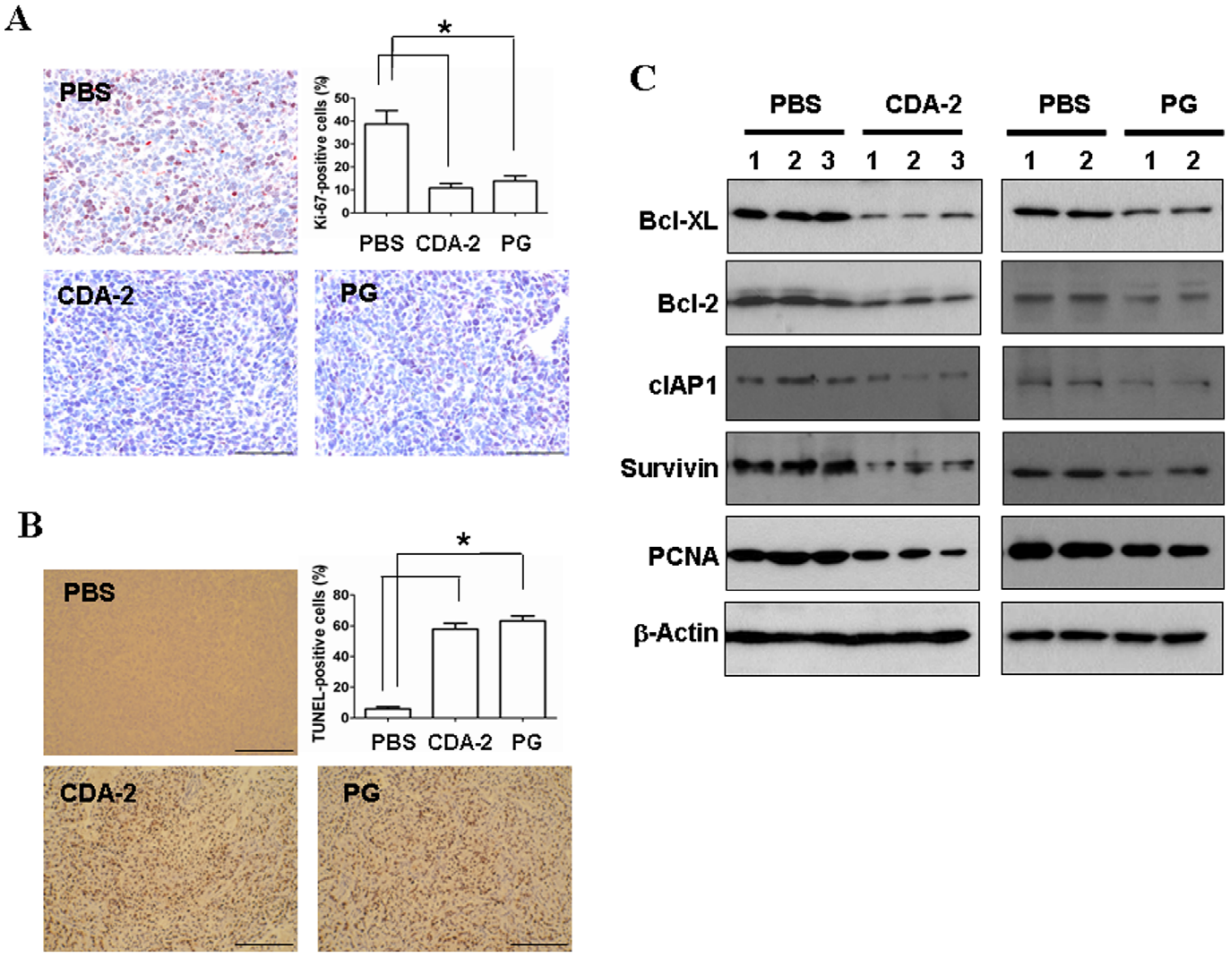
CDA-2 blocks tumor proliferation, enhances tumor apoptosis. (A) Tumor bearing mice were treated by 2000 mg/kg CDA-2 or 800 mg/kg PG for 5 days, and lungs were removed, fixed and paraffin embedded. Paraffin-embedded tumor-bearing lung sections were examined by immunostaining with anti-Ki-67-antibodies to detect proliferating cells. Bar  = 100 µm. Results are means ± SEM, n = 5, significant difference,* p<0.05. (B) Apoptotic cells were identified by TUNEL staining. Bar  = 200 µm. (C) Excised lung tumors were analyzed for the expression of indicated proliferative and apoptotic proteins by immunoblot analysis. The expression of β-actin was used as internal control for the amount of proteins.

Next, to investigate whether CDA-2-induced change in lung tumour burden was due to altered cell proliferation or apoptosis, we examined cell proliferation by immuno-histochemical analysis of Ki-67 and cell apoptosis by in situ terminal-transferase dUTP-mediated nick end labeling (TUNEL) assay. 2×10^5^ LLC cells were injected into mice and 14 days late 2000 mg/kg CDA-2, 800 mg/kg PG or PBS was administered as above for 5 days. Consistent with the changes in lung tumour burden, Ki-67-positive cells were lower in CDA-2 or PG-treated tumors as compared to tumors of PBS-treated animals (Fig. 3A). Conversely, apoptosis was significantly upregulated after CDA-2 or PG treatment, whereas very little apoptosis was seen after PBS treatment (Fig. 3B).

We examined some proteins and genes known to be involved in cell proliferation and apoptosis. Tumors were carefully microdissected using needles from lungs, lysed, and examined by immunoblotting. In contrast to PBS treatment group, expression of the antiapoptotic proteins Bcl-2, Bcl-XL, cIAP1, and Survivin were strongly reduced in response to CDA-2 treatment (Fig. 3C). CDA-2 treatment also decreased the expression of proliferating cell nuclear antigen (PCNA), another S phase marker of cell cycle (Fig. 3C). Immunoblot analysis of tumor lysates of mice also revealed downregulation of Bcl-2, Bcl-XL, cIAP1, and Survivin as well as PCNA in lung tumors after PG treatment (Fig. 3C). These results correlate with those observed by the analysis of Ki-67 and TUNEL.

### CDA-2 Inhibits NF-κB Activation and Pulmonary Inflammation in the Lung of Mice

**Figure 4:**
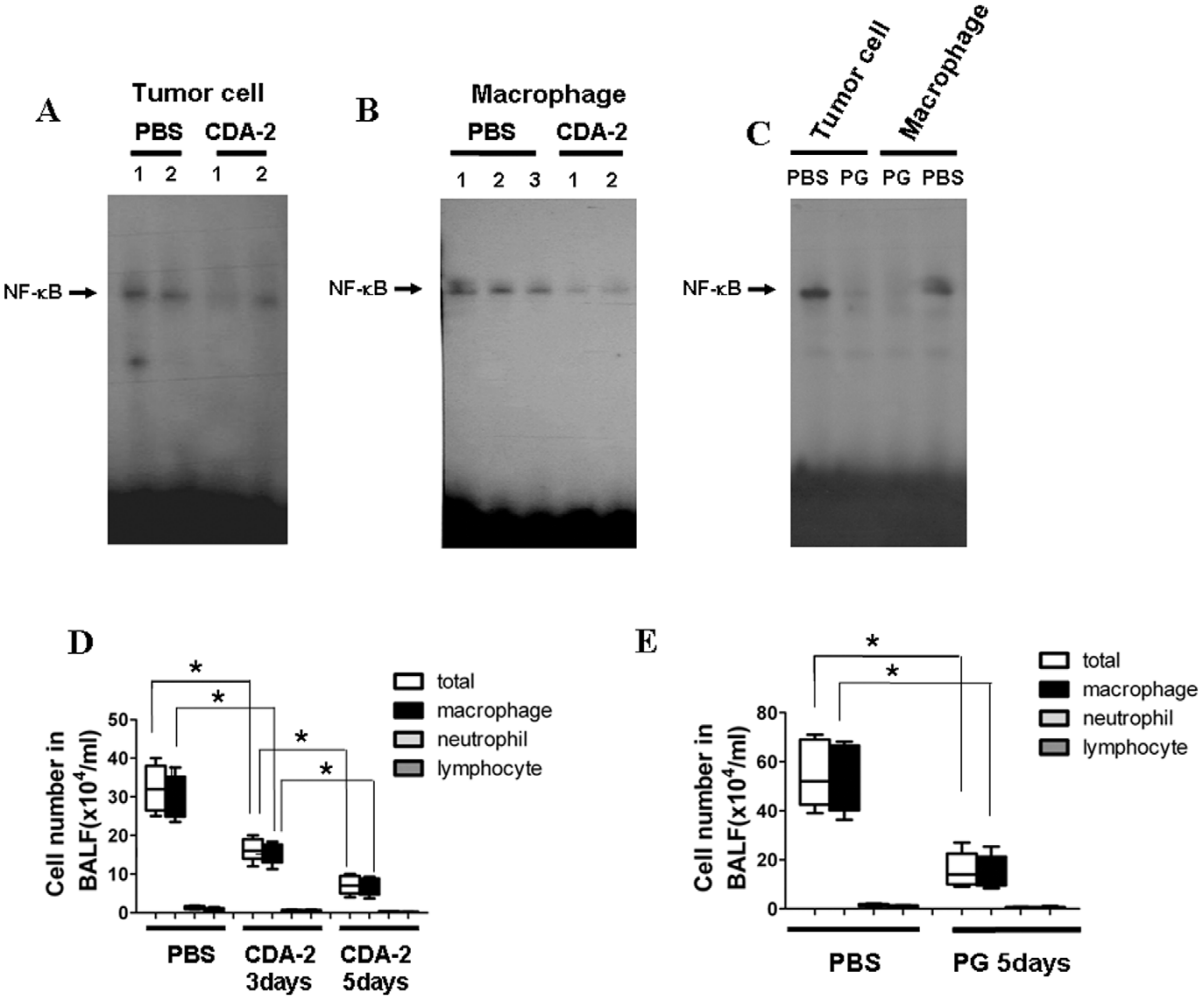
CDA-2 inhibits NF-κB activation and reduces leukocyte populations in BALF. EMSA of nuclear extracts isolated from tumor cells and alveolar macrophages showing nuclear binding of NF-κB after 5 days 2000 mg/kg CDA-2 (A and B) or 800 mg/kg PG (C) treatment. Total cell number and leukocyte population in BALF collected from C57BL6 mice 3 or 5 days 2000 mg/kg CDA-2 (D) and 800 mg/kg PG (E) or PBS treatment. Cellular composition was determined using cytospin preparations. Results are means ± SEM, n = 5, significant difference,* p<0.05.

It has been shown that NF-κB activation plays a pivotal role in regulation of inflammatory and immune response, apoptosis, and oncogenesis which is associated with inflammation-promoting tumor growth [19], [20]. To elucidate the mechanisms of tumor-inhibiting effect of CDA-2, we first compared the NF-κB activation of dissected cancer from mice with CDA-2, PG or PBS treatment. Of note, resected tissue contained tumor tissue including tumor and inflammatory cells. Nuclear extracts were prepared and NF-κB activation examined by EMSA. As shown in Fig. 4A, CDA-2 treatment significantly decreased NF-κB DNA binding activity compared with the control after 5 days 2000 mg/kg CDA-2 treatment. Importantly, NF-κB DNA binding activity also were strongly inhibited in response to CDA-2 treatment in alveolar macrophages of bronchoalveolar lavage fluid (BALF) (Fig. 4B). Next, treatment with 800 mg/kg PG for 5 days in tumor-bearing mice also effectively inhibited the NF-κB DNA binding activity both in tumor cells and alveolar macrophages (Fig. 4C). This result suggests that PG has similar effect in inhibiting the NF-κB activation.

**Figure 5:**
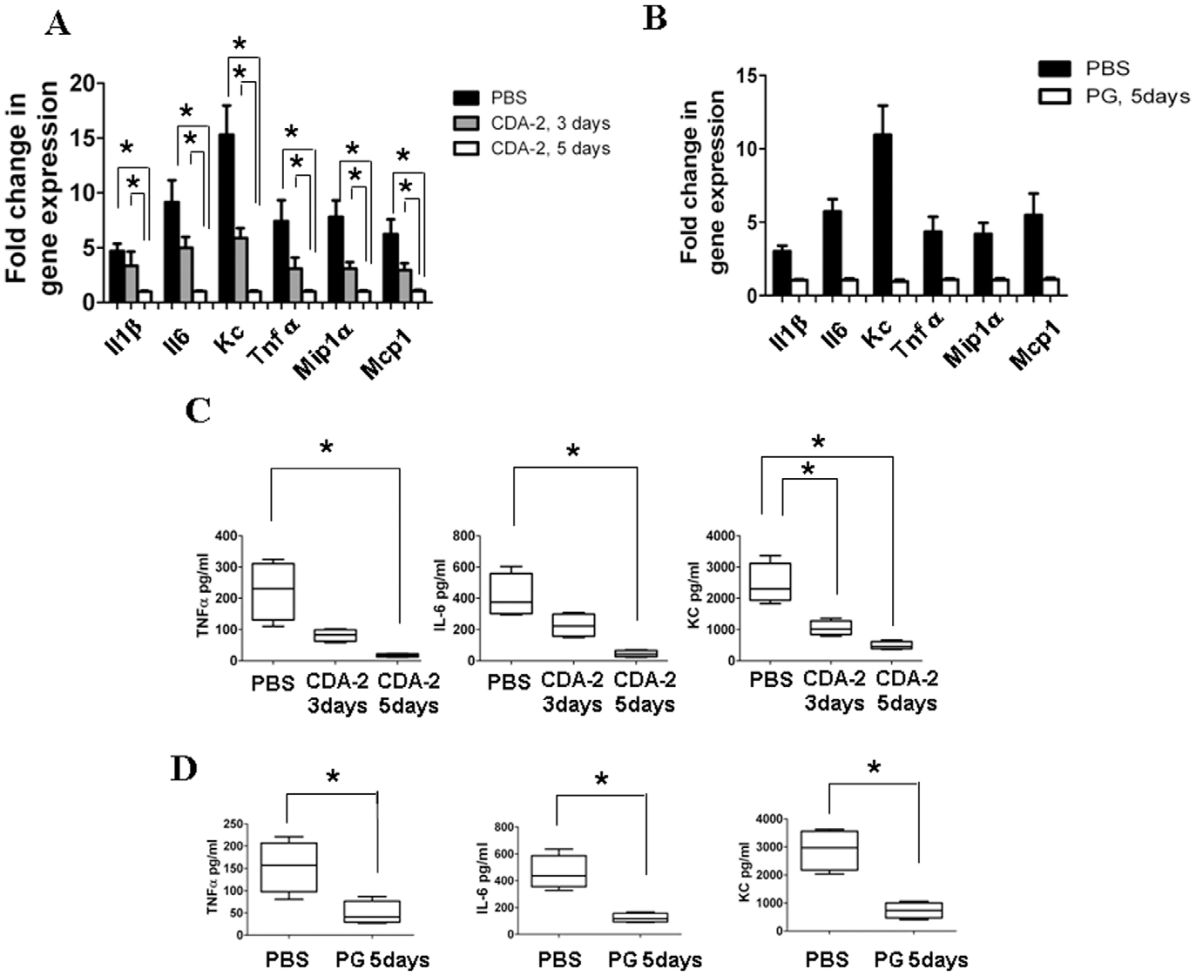
CDA-2 decreases pulmonary inflammation. Induction of inflammatory cytokine and chemokine mRNA in homogenates of 3 or 5 days 2000 mg/kg CDA-2 (A) or 800 mg/kg PG (B) treated lungs was measured by real time PCR. Results are means ± SEM, n = 5, significant difference,*p<0.05. Concentration of inflammatory cytokines in BALF of 3 or 5 days 2000 mg/kg CDA-2 (C) or 800 mg/kg PG (D) treated mice was evaluated by ELISA. Results are means ± SEM, n = 5, significant difference,*p<0.05.

Next, we asked whether the CDA-2-induced inactivation of NF-κB in myeloid cells changes the inflammatory situation in lungs. We characterized inflammatory cells and mediators in lungs of mice subjected to mice cancer model. Total cell number and absolute numbers of macrophages, neutrophils, and lymphocytes in BALF were significantly decreased 3 and 5 days after 2000 mg/kg CDA-2 treatment (Fig. 4D) or 5 days after 800 mg/kg PG treatment (Fig. 4E). CDA-2 or PG treatment effectively reduced the expression of various inflammatory cytokine and chemokine mRNAs, such as Il1b, Il6, Kc, Tnfα, Mip1α, and Mcp1 in the lung (Fig. 5A,B). CDA-2 or PG treatment also decreased secretion of TNF-α, IL-6, and KC by lung cells (Fig. 5C,D). Theses results suggested that reduction of inflammatory reaction by inhibition of NF-κB activation, are likely to be a major tumor-inhibiting mechanism of CDA-2 and PG.

### CDA-2 Inhibits TLR2 Signaling in Bone-marrow-derived Macrophages

**Figure 6:**
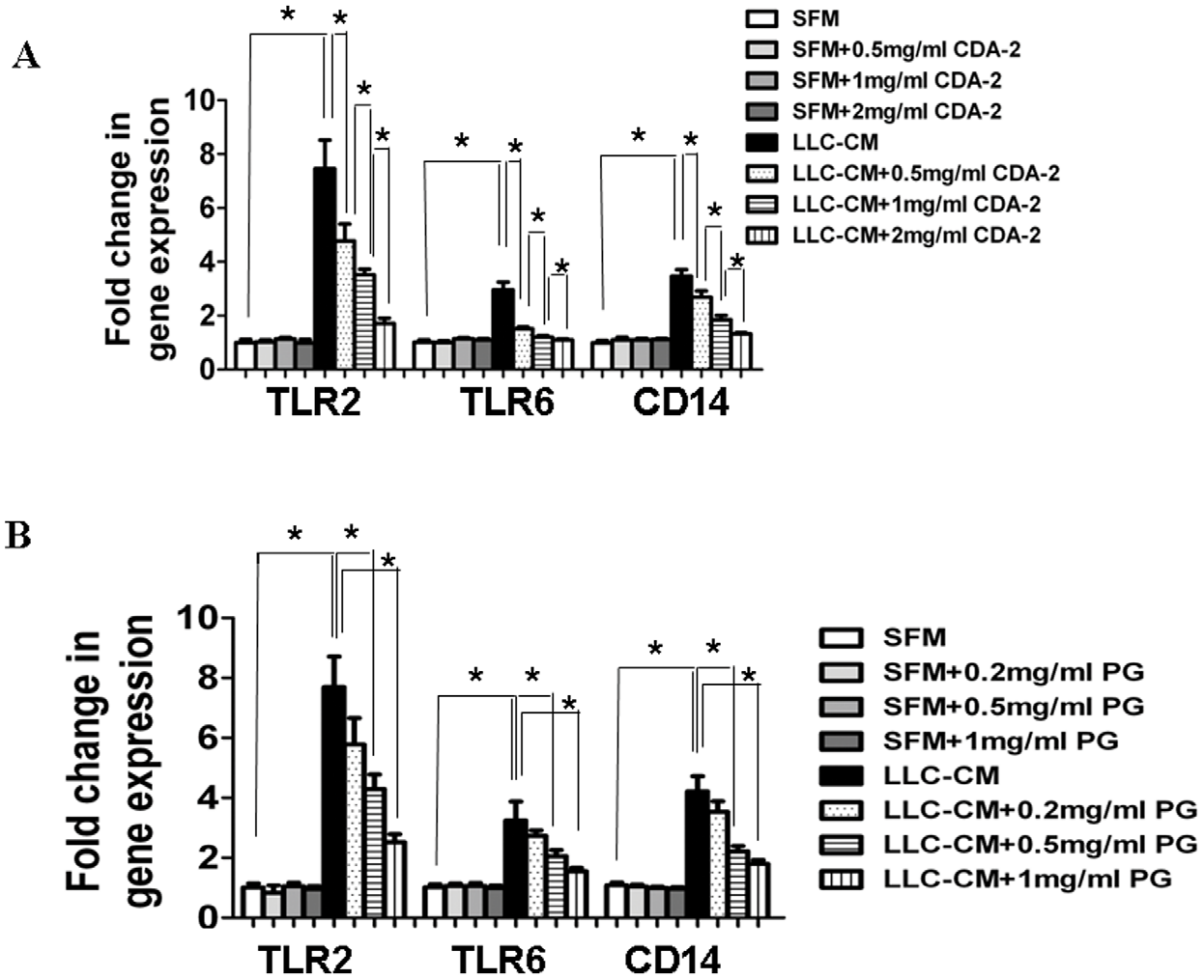
CDA-2 inhibits LLC-CM-induced activation of TLR2 signaling in BMDMs. BMDMs were treated for 24 h by serum-free DMEM (SFM) or LLC-CM or combination with CDA-2 (A) or PG (B). Total RNAs were isolated from BMDMs, and gene expression was assessed by real-time PCR. Results are mean fold change ± SEM, n = 3, significant difference, * p<0.05.

Previous studies have showed that Toll-Like Receptors (TLRs)-mediated signaling favor the activation of NF-κB, leading to a pro-inflammatory response [21]. To address whether CDA-2 inhibited NF-κB through TLR signaling pathway, we examined the expression of TLR family members in bone-marrow-derived macrophages (BMDM) that were stimulated by serum-free conditioned medium from LLC cells (LLC-CM). LLC-CM significantly induced expression of TLR2 and its coreceptor TLR6 and CD14 (Fig. 6A,B), but not TLR1, TLR3, TLR4, and TLR9 in BMDM (data not shown). Importantly, addition of CDA-2 or PG significantly suppressed the expression of TLR2, TLR6, and CD14 in a concentration-dependent manner (Fig. 6A,B). Incubation of BMDM with CDA-2 or PG alone had no effect on TLR2, TLR6, and CD14 expression (Fig. 6A,B). These results suggested that TLR2 signiling could act as mediator and contributes to NF-κB inactivation by CDA-2 and PG.

### Inhibition of CDA-2 to NF-κB Activation was Abrogated by Over-expression of TLR2

**Figure 7:**
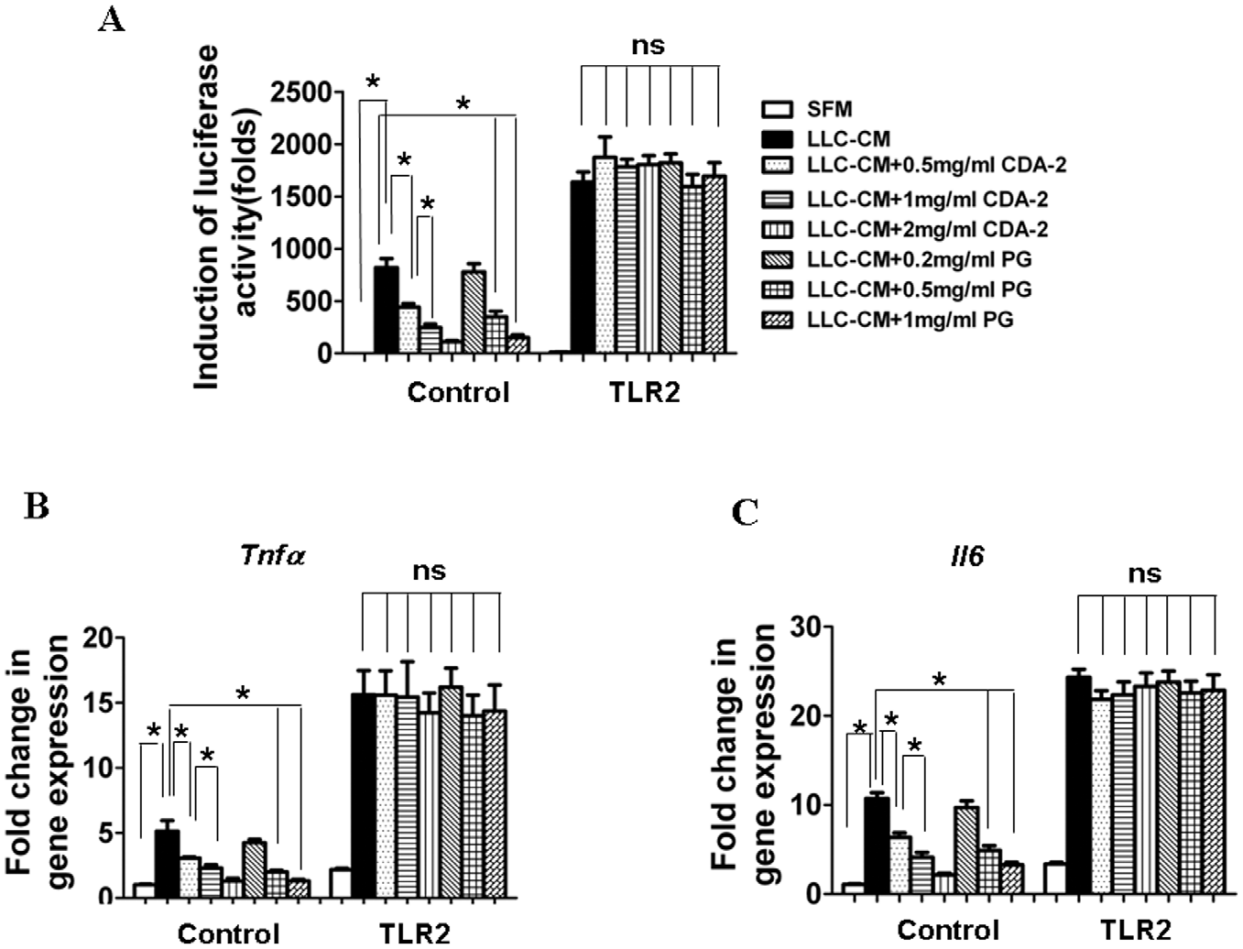
Over-expression of TLR2 abrogates CDA-2-induced inactivation of NF-κB. (A) BMDMs were co-infected with TLR2 and NF-κB luciferase reporter gene adenoviral constructs. 24 hours after infection, cells were treated with SFM or LLC-CM and/or CDA-2 or PG as indicated. Luciferase activities were determined 24 h after the treatment. Data are shown as mean ± SEM fold relative to control. n = 3, significant difference,*p<0.05; ns, not significant. (B) Induction of inflammatory cytokine mRNA in infected BMDMs as above described. Results are mean fold change ± SEM, n = 3, significant difference, *p<0.05; ns, not significant.

To further evaluate TLR2 signaling-mediated NF-κB activation was regulated by CDA-2, we performed NFκB–driven luciferase reporter gene assay. Recombinant adenoviral vectors were generated encoding TLR2 expressed in BMDM. TLR2 or control vector infected BMDM were infected with NF-κB luciferase reporter plasmid adenovirus. Both of infected BMDMs treatment with LLC-CM resulted in significant increases in the binding of NF-κB to its DNA consensus sequence, as displayed by an increase in luciferase activation (Fig. 7A). TLR2 infected BMDM showed significant baseline activations of this transcription factor and higher values by LLC-CM compared with the control values (Fig. 7A). Treatment of CDA-2 or PG caused significant decrease of LLC-CM induced NF-κB transactivation in control infected BMDM, whereas there is no change on reporter activity by CDA-2 or PG in TLR2 infected cells (Fig. 7A). Consistent with the NF-κB transactivation results, these constructs also produced similar effects on expressions of TNFαand IL-6 (Fig. 7B). Thus, TLR2 expression inhibition by CDA-2 and its component PG is required for inactivation of NF-κB in myeloid cells.

## Discussion

The main finding of the present study is that CDA-2, a urinary preparation, inhibits lung tumor growth via a myeloid cell intermediate. CDA-2 reduces the inflammation in lung through suppression of NF-κB activation in myeloid cells associating with modulation of TLR2 signaling. The main constituent of CDA-2, PG, is likely to play a pivotal role to anti-tumor effect of CDA-2.

This study directly tested the important tumor inhibitory effect of CDA-2 by using experimental lung tumor models. Previous studies had shown that CDA-2 is of potential value as anti-cancer agent [3], [7]. CDA-2 has been studied and shown to inhibit the growth of human breast cancer cells, glioma cells, and human leukemia cells *in vitro* and *in vivo* [3], [7]. Clinically, CDA-2 showed significant effects in improving the chemotherapy responses in glioma, hepatoma, non-small-cell lung cancer, and patients with breast cancer [7]. PG is a major bioactive constituent in CDA-2. Previous studies suggest that PG has a potential tumor inhibitory effect, and it also is an important component of antineoplaston AS2-1, a mixture of sodium salts of phenylacetic acid and PG, which is an anti-tumor drug [3], [22]. The present data first confirm that CDA-2 treatment directly results in a growth arrest of lung tumor and an extended life span in mice indicating the potent anti-tumor activity of CDA-2 in inhibiting tumor growth in a dose-dependent manner. Both proliferation-inhibition and apoptosis-inducing effects were observed in lung tumors, as demonstrated by immunohistochemistry and western blotting analysis. PG also exhibits a direct effect on the lung tumor growth, and has similar results of tumor inhibition as CDA-2. These results suggested that CDA-2 might be a potential therapeutic remedy for the treatment of lung cancer, and PG is believed to contribute the major bioactivity of CDA-2 due to high percent (41%) and clear tumor suppression effect.

The tumor microenvironment plays a critical role in tumor initiation and promotion and contains immune cells and connective tissue, such as fibroblasts, endothelial cells, pericytes, and mesenchymal cells [23]. The most frequently found immune cells within the tumor microenvironment are tumor-associated macrophages (TAMs). TAMs mostly promote tumor growth and may be obligatory for angiogenesis, invasion, and metastasis by release of inflammatory cytokines and chemokines, and their presence in lung cancer has been correlated with poor prognosis of lung cancer patients [24], [25] and other outcomes such as increased microvessel count [26]. As parts of positive feed-forward loops, chemokines produced by TMAs attract additional immune/inflammatory cells including macrophages to tumor microenvironment [27]. Our data shows treatment of CDA-2 or PG results in a decrease of total cells in BALF, which mostly affected macrophages, reduced inflammation. Thus, it is likely that CDA-2 inhibits lung tumor growth through suppression of the population of immune/inflammatory cells and inflammation in lung.

Activation of NF-κB is responsible for the induction of a variety of target genes that are important for tumorigenesis [28], [29]. NF-κB activation in inflammatory cells controls the production of pro-inflammatory cytokines, including TNFα, IL-1, IL-6, and IL-23, which mediate tumor promotion and progression, as well as NF-κB activation in tumor cells [8], [11]. NF-κB activation also is found in tumor cells where it regulates cell proliferation, survival, angiogenesis, invasion, and metastasis [30]–[32]. The promoting effect of NF-κB activation on lung carcinogenesis has been already shown with different approaches and reported by different groups, and depletion of myeloid cellular or epithelial NF-κB appear to have an effect in the reduction of lung tumorigenesis [9], [33]–[36]. Recently, CDA-2 was found to inhibit activation of NF-κB in human leukemia and myelodysplastic syndromes (MDS) cell lines in vitro through an impact on NF-κB nuclear translocation by affecting IkBα phosphorylation and degradation [3], [6]. In addition, a recent study suggests that sodium phenylbutyrate, the precursor of PG, can suppress both activation of NF-κB and expression of proinflammatory molecules [37]. These observations led us to examine whether the growth inhibition of lung tumor by CDA-2 is involved in the regulation of NF-κB. We found CDA-2 and PG significantly reduced NF-κB DNA-binding activity in cancer cells and alveolar macrophages and decreased inflammation, suggesting that inactivation of NF-κB by CDA-2 may contribute to the regression of lung tumor. Karin’s group found that IKKβ ablation in myeloid cells reduces the CS induced inflammatory response in the lung and abrogates lung tumor development [9]. We also confirmed the important role of NF-κB inactivation in myeloid cells to abrogation of lung tumor using mice lacking myeloid RelA/p65 in another paper [38]. It has been reported that inhibition of NF-κB in tumor cells including lung cancer, breast cancer, colon cancer, pancreatic cancer, and various types of leukaemia also results in abrogation of proliferation and in increased apoptosis, indicating the crucial role of NF-κB in tumor cell proliferation and survival [32], [39]. Therefore, we could not rule out the proliferation-inhibition and apoptosis-inducing effects of CDA-2 to lung tumor cells directly. Confirmatory treatment experiments of CDA-2 to lung tumor cells directly are ongoing and will be described in a future publication.

TLRs that is mostly expressed on myeloid cells, play a critical role in the innate immune response, they contribute to the activation of NF-κB and induction of inflammatory factors [21], [40]. TLR2 knockout mice exhibited significantly greater survival than WT mice after LLC inoculation and their lungs contained fewer and smaller tumor nodules [41]. Recent studies have demonstrated that LLC cells-produced factors such as versican is necessary for tumor growth and metastasis, a process that depends on TLR2-mediated myeloid cell activation [41], where activation of NF-κB results in inflammatory factors TNFα, IL-6 production [42], [43]. Versican as a macrophage activator that acts through activating TLR2 and its coreceptors TLR6 and CD14, inducing TNFα production [41]. Ours results indicate that the inhibition of NF-κB activation by CDA-2 and PG rely on TLR2. TLR2:TLR1 or TLR2:TLR6 dimers activate NF-κB by recognizes various bacterial components, including peptidoglycan, lipopeptide and lipopetide of Gram-positive becteria and mycoplasma lipopeptide [40], [41]. In the present study, we indicate that TLR6 but not TLR1 as a co-receptor of TLR2 mediates the effect of NF-κB inactivation by CDA-2.

In conclusion, we reported that CDA-2 is very effective at growth inhibition in mouse lung tumor associated with proliferation-inhibition and apoptosis-inducing effects *in vivo*. Suppression of the population of immune/inflammatory cells and inflammation in lung by inhibition of NF-κB inactivation is likely to be a major tumor-reducing mechanism of CDA-2. Here we demonstrate that the decrease of TLR2 signaling activation by CDA-2 is an important contributor to the NF-κB inactivation. These results strongly suggest that CDA-2 is a potential agent for the treatment of lung cancer and warrants further study.
